# Spontaneous Pneumomediastinum/Pneumothorax in Patients With COVID-19

**DOI:** 10.7759/cureus.8996

**Published:** 2020-07-03

**Authors:** Bianka Eperjesiova, Eric Hart, Mohamed Shokr, Prabhat Sinha, Gary T Ferguson

**Affiliations:** 1 Pulmonary and Critical Care Medicine, Ascension Providence Hospital, Southfield, USA; 2 Cardiology, Detroit Medical Center, Detroit, USA; 3 Pulmonary and Critical Care Medicine, Pulmonary Research Institute of Southeastern Michigan, Farmington Hills, USA

**Keywords:** pneumothorax, pneumomediastinum, covid-19, mechanical ventilation

## Abstract

No spontaneous air leak case series have been described in the severe acute respiratory syndrome coronavirus 2 (SARS-CoV-2) patient population thus far. We described seven spontaneous air leak cases we found in our coronavirus disease 2019 (COVID-19) positive 976-patient cohort. Five out of seven patients eventually required mechanical ventilation, and one of these patients died. All of our patients who demonstrated radiological air leaks after intubation died. No other precipitating factors offered in the literature thus far played a role in our patient population. We presume that acute lung injury leading to SARS-CoV-2 with associated acute respiratory distress syndrome (ARDS) predisposes patients to this rare complication.

## Introduction

Spontaneous air leak is the travel of free air following distended ruptured alveoli via peribronchovascular sheaths into the mediastinum or the pleural space [1]. It can occur in the setting of obstructive or restrictive lung disease exacerbation, although it has been observed in hyperventilation states such as infections, vomiting, diabetic ketoacidosis, athletic activities, or inhalational drug abuse [2]. Air leak in hospitalized severe acute respiratory syndrome coronavirus 2 (SARS-CoV-2) patients has been sparsely published as case reports, however, the presence of non-invasive ventilation and positive airway pressure were reported prior to this finding [3-4]. Additionally, there is no reported incidence found in the literature. Studies of a severe acute respiratory syndrome with SARS did identify air leak as a frequent complication, often with no relation to intubation or positive pressure ventilation [1-5].

## Case presentation

Our institution cared for 976 coronavirus disease 2019 or COVID-19 (SARS-CoV-2) patients between March 8 and April 15, 2020. Of these, we found 20 cases of air leak; three traumatic/post-procedure, 10 post-intubation/mechanical ventilation, and seven spontaneous (five cases of pneumomediastinum and two isolated pneumothorax). The demographic, clinical, laboratory, and imaging data of the seven patients who developed a spontaneous air leak are provided in Table 1.

**Table 1:** Demographic, laboratory, and radiological data for seven COVID-19 patients with spontaneous air leak

	Patient 1	Patient 2	Patient 3	Patient 4	Patient 5	Patient 6	Patient 7
Timing in relation to invasive ventilation	Never Intubated	Before	Before	Never Intubated	Before	Before	Before
Pneumomediastinum (PM)/Subcutaneous emphysema (SE)/Pneumothorax (PTX)	PM, SE extending into the neck	PM, SE	PM, SE	PM, Left PTX	PM, SE	Right PTX	Right PTX
Days from Admission to Event	0	10	15	0	0	18	0
Cough	No known cough	Mild cough	Forceful	Forceful	Forceful	Forceful	Forceful
Maximum Respiratory Rate	24	18	30	20	17	28	32
Smoking Status	Active	Never	Never	Never	Never	Never	Never
History of Pulmonary Disease	Emphysema	None	Asthma	None	Asthma	None	None
Lactate mmol/L (Initial)	4.3	1.8	2.6	N/A	3.3	2.4	3.4
Other Computed Tomography Findings	Diffuse Airspace Disease with Ground Glass Opacities (GGOs)	Diffuse Airspace Disease with GGOs	Diffuse Airspace Disease with GGOs	Diffuse Airspace Disease with GGOs	Diffuse Airspace Disease with GGOs	Diffuse Airspace Disease with GGOs	Diffuse Airspace Disease with GGOs and Right Lower Lobe (RLL) Consolidation
Chest Tube Placement	No	No	No	Yes	No	Yes	Yes
Air Leak Radiological Resolution	Yes	No	Yes	Yes	No	Yes	Yes
Length of stay (days)	14	15	25	22	40	36	15

**Figure 1:**
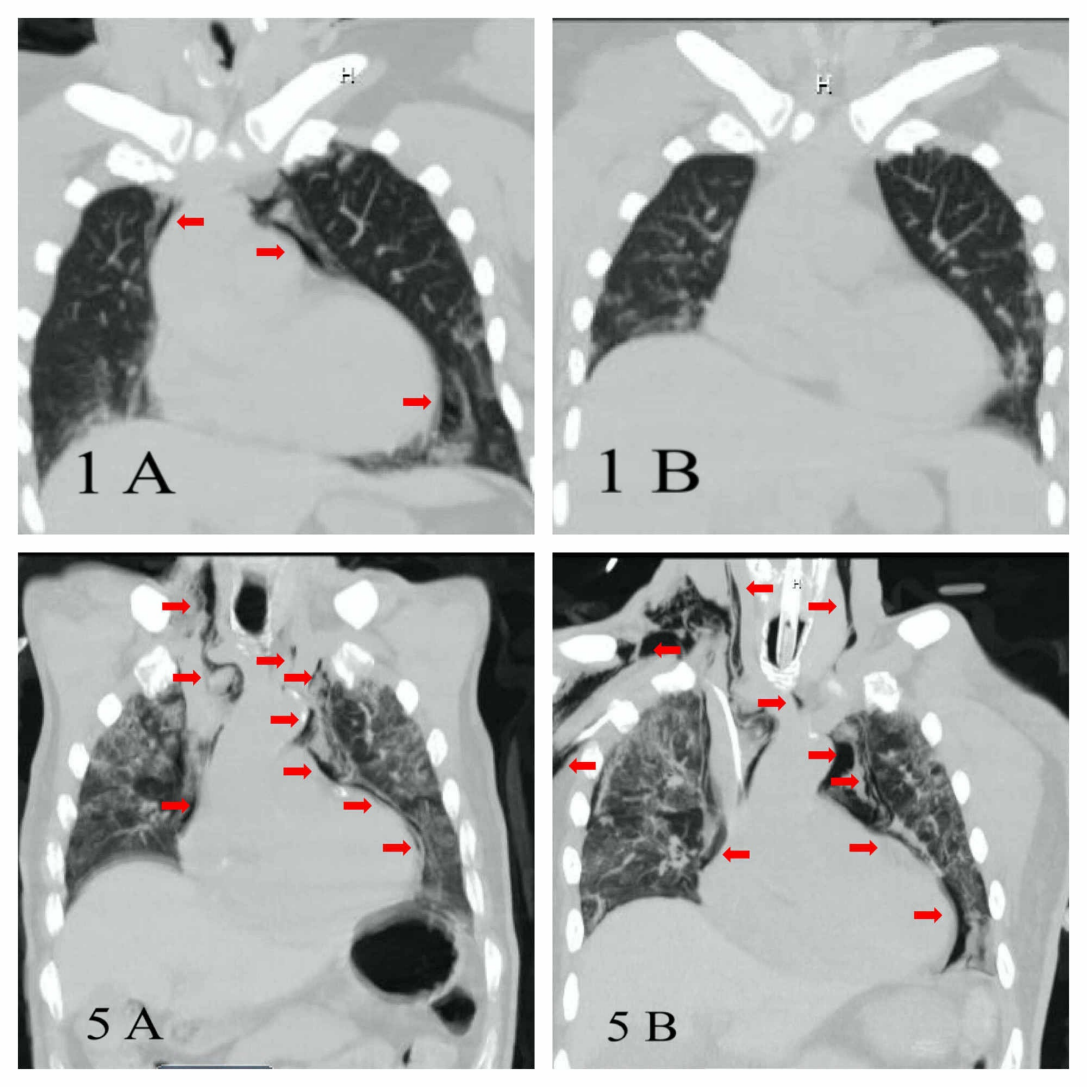
Computed tomography (CT) of the chest for patient 1 and patient 5 showing initial and follow-up imaging 1A - CT chest with pneumomediastinum extending into the subcutaneous emphysema in the neck and bilateral ground glass opacifications, 1B - CT chest showing resolution of the air leak, 5A - CT chest with pneumomediastinum tracking into the neck, 5B - CT chest post-intubation with severe worsening of the air leak

A typical clinical scenario is described as follows. A 54-year-old woman, with a history of hypertension presented with altered mental status. She was hemodynamically stable but with mild hypoxemia. She was COVID-19 positive, had elevated inflammatory markers, and acute renal failure requiring dialysis. Computed tomography (CT) scan showed changes consistent with acute respiratory distress syndrome (ARDS) plus pneumomediastinum with subcutaneous emphysema extending into the neck (Figure 1 - 1A). The patient was treated medically, and no mechanical ventilation was required. Her hospital course was complicated by a bleeding duodenal ulcer, ischemic stroke, and fungemia. Fourteen days later, CT showed a resolution of pneumomediastinum (Figure 1 - 1B). She was discharged to subacute care. Similarly for patient 5, CT was performed (Figure 1 - 5A, 5B).

The patient was treated medically, and no mechanical ventilation was required. Her hospital course was complicated by a bleeding duodenal ulcer, ischemic stroke, and fungemia. Fourteen days later, CT showed the resolution of pneumomediastinum (Figure 1 - 1B). She was discharged to subacute care.

## Discussion

Radiological evidence of spontaneous air leak was found in seven patients (incidence 0.72%) with event onset 9±9 days (mean±SD) from COVID-19 symptom onset and 6±8 days (mean±SD) from admission. Our cohort had no gastrointestinal/thoracic/vascular procedures or chest compressions during hospitalization. Five out of seven patients required intubation, but all pneumomediastinum/pneumothorax events occurred before intubation. No difference in age, gender, body mass index, or medical histories as compared to other COVID-19 patients was noted except for three out of seven patients having an obstructive lung disease. One out of seven spontaneous air leak patients expired. Five out of six discharged patients had resolution of their air leak. All 10 patients with an air leak after intubation expired. CT chest of patient number 5 from our series, who expired, is depicted in Figure 1 - 5A pre-intubation, with progression in Figure 1 - 5B post-intubation.

## Conclusions

We presume that COVID-19 infection leading to acute lung injury with associated ARDS might be associated with a spontaneous air leak, and further investigations are warranted to delineate mechanisms and impact on outcomes.
